# ﻿*Cardaminezhangjiajieensis*, a new species of Brassicaceae in China

**DOI:** 10.3897/phytokeys.248.119678

**Published:** 2024-11-04

**Authors:** Jia-Lu Li, Cheng Zhang, Yi He, Quan-Ru Liu

**Affiliations:** 1 Key Laboratory of Biodiversity Science and Ecological Engineering, Ministry of Education, College of Life Sciences, Beijing Normal University, Beijing 100875, China Beijing Normal University Beijing China; 2 Shenzhen Key Laboratory for Orchid Conservation and Utilization, and Key Laboratory of National Forestry and Grassland Administration for Orchid Conservation and Utilization, Shenzhen 518114, China Shenzhen Key Laboratory for Orchid Conservation and Utilization, and Key Laboratory of National Forestry and Grassland Administration for Orchid Conservation and Utilization Shenzhen China; 3 The National Orchid Conservation Center of China and the Orchid Conservation & Research Center of Shenzhen, Shenzhen, 518114, China The National Orchid Conservation Center of China and the Orchid Conservation & Research Center of Shenzhen Shenzhen China

**Keywords:** Flora of Hunan, morphology, new species, phylogeny, taxonomy

## Abstract

*Cardaminezhangjiajieensis*, a new species from Hunan Province, China, is described and illustrated. *Cardaminezhangjiajieensis* is similar to *C.circaeoides*. From the latter, *C.zhangjiajieensis* is readily distinguished by the terminal leaflet of the cauline leaf 4.5–7 cm (vs. 2.5–4.5 cm), mostly round or oblong (vs. oblong-oval to triangular-oval), margin undulating sinuses (vs. crenate or subentire), seed apically narrow wing (vs. wingless).

## ﻿Introduction

*Cardamine* L. (Brassicaceae) is a cosmopolitan genus with over 280 species (Marhold et al. 2021), mainly distributed in the temperate regions. According to the *Flora of China*, there are 48 species of *Cardamine* in China, 24 of which are endemic (Zhou et al. 2001). Since 2001, new species and distribution records of Chinese *Cardamine* have been published continuously (Marhold et al. 2007; Al-Shehbaz and Boufford 2008; Al-Shehbaz 2015a, 2015b; An et al. 2016). To date, 61 species of *Cardamine* have been reported in China, 31 of which are endemic.

We examined literature and specimens of *Cardamine*. Field surveys were conducted in Hunan, Sichuan, Jilin, Hebei, Yunnan from 2021 to 2023. During the specimens examination of the genus *Cardamine*, the author picked out one specimen which was identified as *Cardaminemacrocephala* Z. M. Tan & S. C. Zhou (Tan and Zhou 1996), *Zhang D. G. 130502028* collected from Zhangjiajie Nature National Reserve, Hunan Province. This name had been recorded as a synonym of *C.circaeoides* Hook.f. & Thomson (Zhou et al. 2001). The morphological characters of the plants on the specimen *Zhang D. G. 13050202*8 are significantly inconsistent with those of *C.circaeoides*, whose leaves are heart-shaped, 1.5–5.5 cm long, margin entire. In contrast, the leaves of *Zhang D. G. 130502028* are oval to nearly round, 3–7 cm long, with wide undulating teeth. Therefore, in order to clarify the taxonomic status of the above two groups, this study conducted field investigations, morphological and systematic analysis. This species, *C.zhangjiajieensis*, is proposed as new to science.

## ﻿Method

### ﻿Sampling and morphological analyses

**Figure 1.**
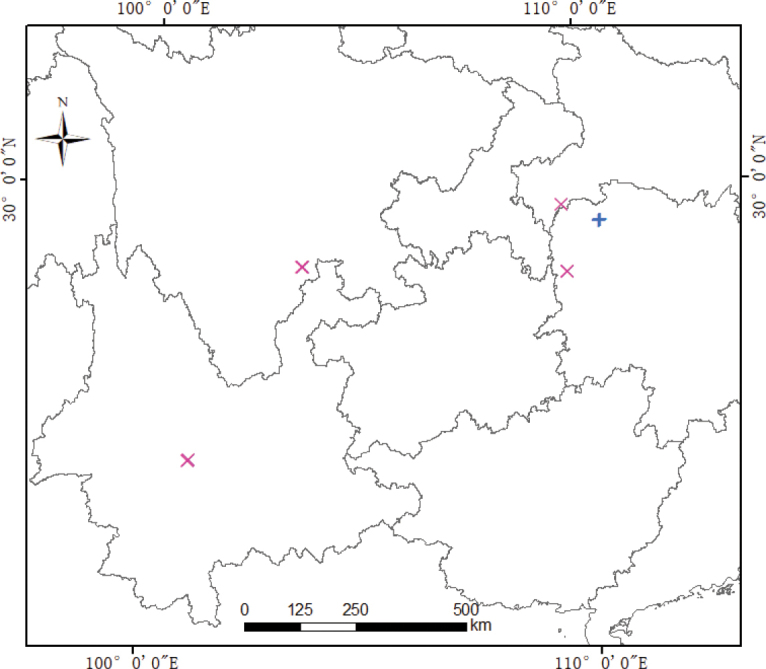
Collection site of *C.zhangjiajieensis*, plus represents *C.zhangjiajieensis* and the cross represents *C.circaeoides*.

Based on the literature examination, more than 5000 specimens from PE, KUN, CDBI, SZ and other herbaria were examined. Field surveys were conducted in Zhangjiajie Nature National Reserve during April 2021, May 2022 and May 2023, specimens’ collection sites are shown in Table 1, Fig. 1.

**Table 1.** Taxa and sample sites.

Taxa and sample sites	No. of individuals	Morphological analyses	Flow cytometric measurements
***C.zhangjiajieensis*** J.L. Li & Q.R. Liu			
China Hu’nan: Zhangjiajie Nature Reserve, Wulingyuan, 29.340766°N, 110.45326°E, 880 m, May 21, 2022, *J.L.Li & C. Zhang BNU2022ZJJ02*	3	√	√
China, Hu’nan: Zhangjiajie Nature Reserve, Wulingyuan, 29.324980°N, 110.433310°E, 600 m, May 21, 2022, *J.L.Li & C. Zhang BNU2022ZJJ06*	5	√	
China, Hu’nan: Zhangjiajie Nature Reserve, Wulingyuan, 29.341153°N, 110.457903°E, 820 m, May 21, 2022, *J.L.Li & C. Zhang BNU2022ZJJ04*	3	√	
China, Hu’nan: Zhangjiajie Nature Reserve, Jinbianxi, 2021, *C. Zhang, zhang670*	9	√	
***C.circaeoides*** Hook.f. & Thomson			
China Yunnan: Ailaoshan, alt. 2523 m, 24.535°N, 101.023198°E, 26 May 2022, *C. Zhang & X.J.Zhao BNU2022ALS01*	12	√	
China Yunnan: Ailaoshan, alt. 2475 m, 24.525649°N, 101.008964°E, 26 May 2022, *C. Zhang & X. J .Zhao BNU2022ALS03*	5	√	
China Sichuan: Yinchanggou Village, alt. 1200 m, 28°30'9.17"N, 103°30'40.83"E, 30 April 2022, *C. Zhang & J.L. Li BNU2022XN01*	9	√	
China Sichuan:Yinchanggou Village, alt. 1200 m, 28°30'0.91"N, 103°29'10.44"E, 30 April 2022, *C. Zhang & J.L. Li BNU2022XN03*	4	√	
China Hubei: Xuan’en, Shadaogou, *C. Zhang, zhang660*	5	√	
China Hu’nan: Jishou, Hangxia Spring, *C. Zhang, zhang551*	2	√	
China Hu’nan: Jishou, Hangxia Spring, *C. Zhang, zhang531*	6	√	
**C.cf.scutata** Thunb.			
**China** Sichuan: Michangshan, alt. 1256 m, 32.633917°N, 106.517870°E, 14 May 2022, *C. Zhang & J.L. Li BNU2022MCS02*	3		√
***C.hupingshanensis*** K.M.Liu, L.B.Chen, H.F.Bai & L.H.Liu			
**China** Hu’nan: Hupingshan April 2023, *BNU2023HPS01*	2		√

Morphology characters of rhizome, stem, leaf, flower and silique have been carefully revalued by Image J. A list of morphological characters and their acronyms used in analyses has been shown in Table 2. The description of character was referred to (Šlenker et al. 2018). 14 unrelated characters were selected, and PCA was performed by Past 3 after standardization by IBM SPSS Statistics (v.27).

**Table 2.** List of morphological characters and their acronyms used in analyses.

Acronym	Description of character
RW	Indument in the plant (0-hairs present, 1-hairs absent)
BL	Length of the terminal leaflet of the basal leaf (mm)
BW/BL	Ratio of the length and width of the terminal leaflet of the basal leaf
BWL/BL	Ratio of the widest part length and length of the terminal leaflet of the basal leaf
BTA	Angle of the apex of the terminal leaflet of the basal leaf (mm)
BBA	Angle of the basal of the terminal leaflet of the basal leaf (mm)
No.BL	Mean number of lateral leaflets of the basal leaf (mm)
BTD	Depth of sinuses of the terminal leaflet of the basal leaf (mm)
CL	Length of the terminal leaflet of the cauline leaf (mm)
No.CL	Mean number of lateral leaflets of the cauline leaf (mm)
CTA	Angle of the apex of the terminal leaflet of the cauline leaf (mm)
CBA	Angle of the basal of the terminal leaflet of the cauline leaf (mm)
CBL/CL	Ratio of the widest part length and length of the terminal leaflet of the basal leaf
CTL	Depth of sinuses of the terminal leaflet of the cauline leaf (mm)
PL	Length of petals (mm)
PW	Width of petals (mm)
SL	Length of sepals (mm)
SW	Width of sepals (mm)
LFL	Length of longer filaments (mm)
SFL	Length of shorter filaments (mm)
PdL	Length of the pedicel of the lowermost siliqua of the main inflorescence (mm)
SqL	Length of the lowermost siliqua of the main inflorescence (mm)

### ﻿Phylogenetic analyses

Fresh plant leaves were collected in the field and quickly dried with silica gel. Plant samples were sent to Beijing Novogene Corporation for quality testing and re-sequencing. The sequencing platform, Illumina HiSeq X Ten and BGI, was used to generate approximately 2–10 GB of data for each sample. The chloroplast genome was assembled from the clean data using GetOrganelle (Jin et al. 2020). Plastid Genome Annotator (PGA) was used to annotate chloroplast genome with *Amborellatrichopoda* Baill. from software as references (Qu et al. 2019). Then, 26 plastid genome sequences were downloaded from NCBI (Table 5), including 23 species of *Cardamine* and 2 species, *Rorippasylvestris* (L.) Besser, *Rorippaindica* (L.) Hiern as outgroup.

The annotated sequences were imported into PhyloSuite (Zhang et al. 2020), the Mafft module was used for sequence alignment (Katoh et al. 2019), and the ModelFinder module was used to calculate the nucleotide substitution model for the aligned sequences. The maximum likelihood (ML) tree was constructed using IQ-TREE (Minh et al. 2020), with the nucleotide substitution model set to GTR+R3+F and a standard bootstrap value of 1000.

### ﻿Flow cytometric measurements and estimation of DNA ploidy levels

Methods referring to Marhold et al. (Marhold et al. 2010) and Kobrlová and Hroneš (Kobrlová and Hrones 2019) measured the nuclear DNA content using flow cytometry. Inferred the DNA ploidy levels within the studied populations based on *Cardamine* species with known ploidy (Suda and Trávníček 2006; Marhold et al. 2021). The relative nuclear DNA content was determined using PI, a DNA intercalating fluorescent dye, with arbitrary units (a.u.) as the unit of measurement. The buffer solution used was LB01. Dehydrated leaves, preserved by drying at 40 °C for 18–24 months, were used for the determination of chromosome ploidy. The sample sources and voucher specimens are presented in the Table 1. In a pre-cooled culture dish, 1–2 mL of LB01 buffer solution and 2 cm^2^ of dry leaves were added. After rapid chopping, the mixture was filtered through a 400-mesh gauze, centrifuged at 4 °C, 3000 rpm for 10 minutes, and the supernatant was discarded. The pellet was resuspended in 600 μL of LB01 buffer solution, followed by the addition of 100 μL of PI solution (50 μg/mL), and stained in the dark for 15 minutes. Ploidy level of the stained cell suspension was determined by flow cytometry (ACEA NovoCyte 3130). Using 488 nm blue light excitation, 10,000 cells were collected at a time. The other samples were determined under the same voltage bar using *C.scutata* (2n = 4x = 32) as the reference for tetraploid.

## ﻿Result

### ﻿Morphological analyses

**Figure 2.**
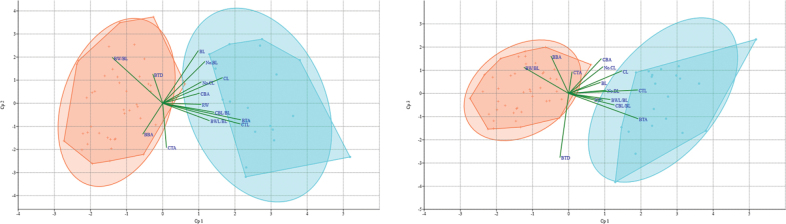
Ordination diagrams of principal component analyses, orange plus refers to *C.circaeoides*, cyan dots refers to *C.zhangjiajieensis*.

The ordination diagrams of PCA based on individual plants (Fig. 2) showed that, the cumulative proportion of eigenvalue of the first five principal component axes exceeds 75%, indicated success of dimension reduction. *C.circaeoides* and *C.zhangjiajieensis* were separated on principal component axis 1 without overlap. The characters that contributed to this axis are the angle of the basal of the terminal leaflet of the basal leaf (BBA), depth of sinuses of the terminal leaflet of the cauline leaf (CTL), ratio of the widest part length and length of the terminal leaflet of the basal leaf (CBL/CL), indicating significant differences in cauline leaf morphology. The principal component axis 2 and axis 3 have overlapping distribution ranges.

### ﻿Phylogenetic analyses

**Figure 3.**
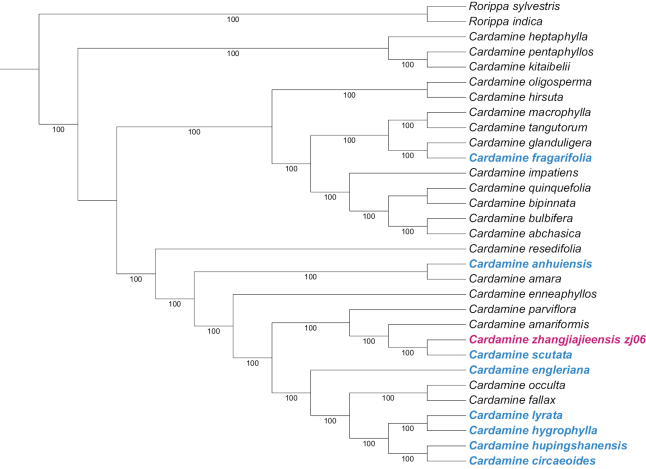
The strict consensus tree resulted from IQ-tree analysis using plastid genome. Bootstrap (BS) are showed below branches. Blue refers to species distributed in Hunan province, rose red refers to the novelty.

The aligned plastid genome dataset included 32 species with 161195 bp characters; 6552 (4.06%) were parsimony informative. As shown in the Fig. 2, the plants used in this study were divided into three branches, *Cardamine* species formed a monophyletic group with moderate support (BS = 1). *C.zhangjiajieensis* and *C.scutata* converged into a single lineage, formed a clade with *C.amariformis* Nakai. in Fig. 3.

### ﻿Flow cytometric measurements and estimations of DNA ploidy levels

As shown in Table 3, the chromosome ploidy was determined by the materials stored for 18–24 months after being dehydrated, and the coefficient of variation was between 6.9% and 7.9%, indicating that dried specimens could be used in this study (Suda and Trávníček 2006). According to the relative DNA content and chromosome number of *C.scutata*, the relative DNA content of *C.zhangjiajieensis* and *C.hupingshanensis* was measured, as shown in the table. The chromosome ploidy of *C.zhangjiajieensis* was estimated to be 2n ≈ 6x.

**Table 3.** Relative genome sizes obtained for *C.zhangjiajieensis*, *C.scutata*, *C.hupingshanensis*, the Relative genome sizes of *C.scutata* refer to (Marhold et al. 2010).

Taxa and Voucher information	Relative genome size in a.u. (arbitrary units); mean (minimum–maximum)	(DNA) Ploidy level	Mean relative genome size per monoploid genome	Variation (%)
*C.zhangjiajieensis*	0.727 (0.670–0.784)	≈6x	-	7.9%
*C.scutata*	0.466 (0.405–0.527)	4x	0.117	6.9%
*C.hupingshanensis*	0.911 (0.842–0.980)	3x	0.304	7.6%

### ﻿Taxonomic treatment

#### ﻿ Cardamine zhangjiajieensis

Taxon classificationPlantaeBrassicalesBrassicaceae

Q.R.Liu & J.L.Li sp. nov.

18D95061-D902-5FD6-868C-8ECCDE2CBE23

urn:lsid:ipni.org:names:77351286-1

Figs 4 , 5

##### Type.

China • Hunan: Zhangjiajie Nature Reserve, Wulingyuan, 29.340766°N, 110.45326°E, 880 m, May 21, 2022, *Li JL, Zhang C, BNU2022ZJJ02* (holotype:BNU0057018, isotype: BNU0057017, BNU0057016).

**Figure 4.**
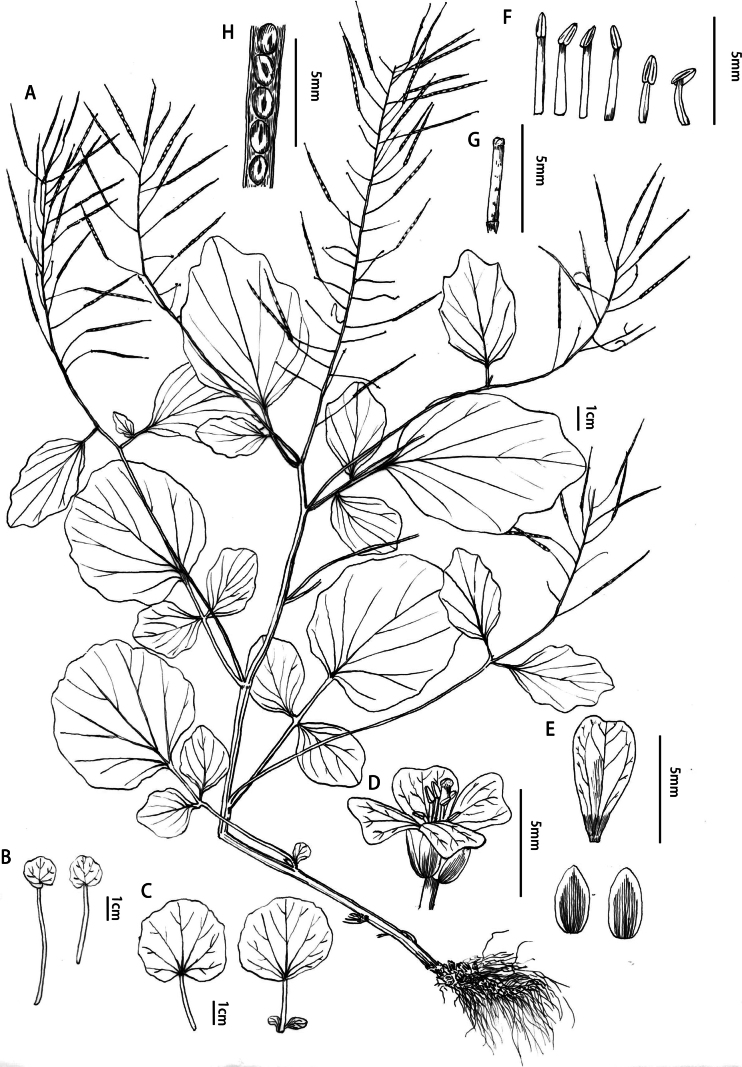
*Cardaminezhangjiajieensis* Q.R.Liu & J.L.Li **A** plants in fruit period **B** new leaves emanating from aerial roots **C** basal leaves **D** flower **E** petal and sepals **F** stamens **G** style **H** siliques and seed, All drawn by Quan-Ru Liu from voucher specimens *Li JL, Zhang C BNU2022ZJJ02* (BNU!) (**A**), *Li JL, Zhang C BNU2022ZJJ04* (BNU!) (**B–H**).

##### Diagnosis.

*Cardaminezhangjiajieensis* is similar to *C.circaeoides*. From the latter, *C.zhangjiajieensis* is readily distinguished by the terminal leaflet of the cauline leaf 4.5–7 cm (vs. 2.5–4.5 cm), lateral leaflets 0–2 pairs (vs. 0–1 pairs), without auriculate petioles (vs. auriculate petioles), mostly round or oblong (vs. oblong-oval to triangular-oval), margin undulating sinuses (vs. crenate or subentire), seed apically narrow wing (vs. wingless).

**Figure 5.**
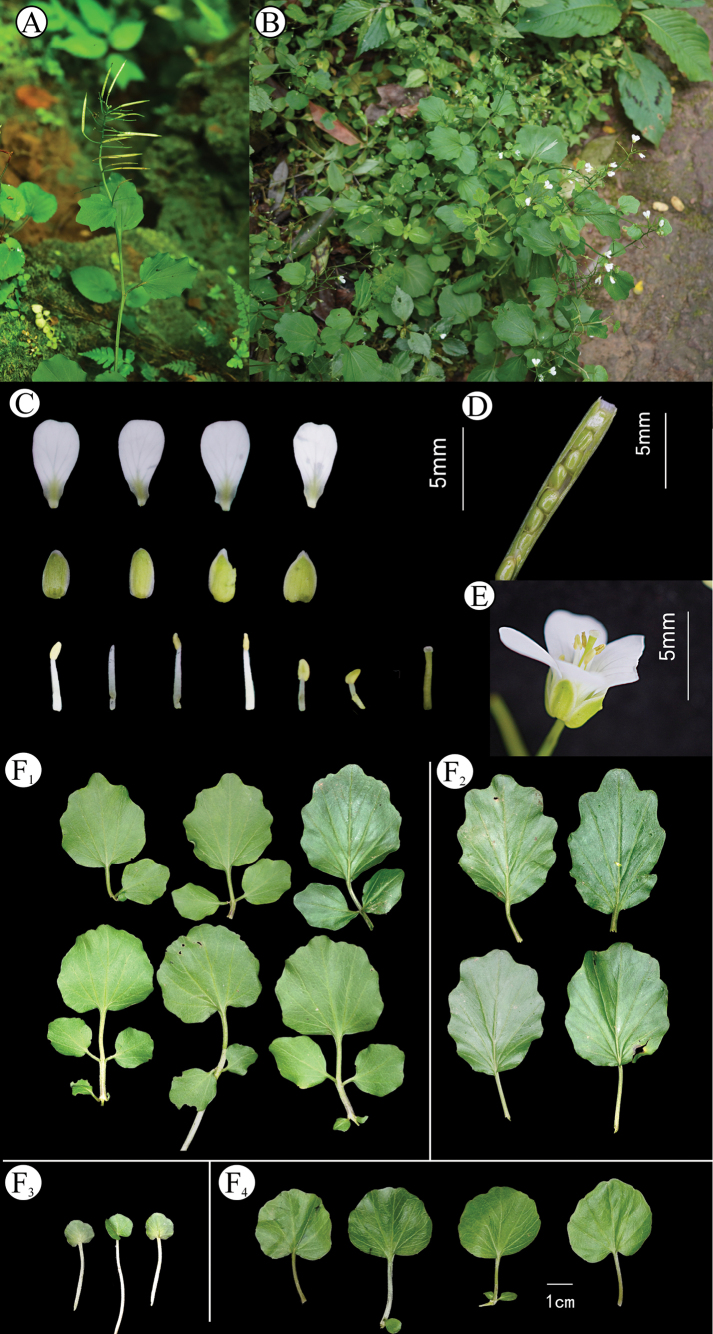
Morphology and habitat of *C.zhangjiajieensis***A, B** plants in flowering and fruit period **C** flower **D** siliques and seed morphology **E** flower (lateral view) **F** leaves, indicating cauline leaves (**F_1_, F_2_**), basal leaves (**F_4_**), and new leaves emanating from aerial roots (**F_3_**). Photographs **A, B, D, E** by Zhang C, **C, F** by Li JL.

##### Description.

Herbs perennial, 15–40 cm tall, glabrous. Rhizomes short, sometimes with a few stolons. Stems erect, simple or branched above middle. Basal leaves not rosette, simple or 2–4 foliolate; petiole 3–6 cm, glabrous; leaf blade or terminal leaflets cordate or ovate, 3–5 cm × 3–5 cm, glabrous, base cordate, sometimes subtruncate, or subcuneate, margin often sinuous, apex obtuse; lateral leaflets (when present) petiolulate or sessile, much smaller than terminal one. Cauline leaves simple or 1 of which 2– or 3–foliolate, petiolate or rarely uppermost subsessile; petiole (0.4–)1–5 (–6) cm; leaf blade similar to that of basal leaves, cordate, sometimes ovate to ovate-lanceolate, 4.5 (–6) × 3(–4) cm. Fruiting pedicels 3–12 (–15) mm, ascending, base sometimes rooting, emitting new plants. Sepals ovate or oblong, 2.8–3.2 mm × 1.4–1.9 mm, glabrous, margin often membranous. Petals white, spatulate, 4.5–6.5 mm × 2.5–3 mm, apex notch. Median filament pairs 3.5–4.5 mm, lateral pair 1.5–2.5 mm; anthers oblong. Siliques linear, 2.5–4 cm; valves torulose, glabrous; style (0.5–)1–2 mm. Seeds brown, ovate or broadly oblong, 1.4–1.6 × 0.9–1.1 mm, with a narrow wing. Fl. and fr. April–Jul. 2n ≈ 6x.

##### Distribution.

Only found in Zhangjiajie Nature Reserve, Hunan Province, grows in wet places of roadside, forest, river sides.

##### Specimens examined.

China • Hunan: Zhangjiajie Nature Reserve, Wulingyuan, 29.324980°N, 110.433310°E, 600 m, May 21, 2022, *Li JL, Zhang C, BNU2022ZJJ06* (BNU, 4 duplicates) • Zhangjiajie Nature Reserve, Wulingyuan, 29.341153°N, 110.457903°E, 820 m, May 21, 2022, *Li JL, Zhang C, BNU2022ZJJ04*, (BNU, 7 duplicates); April 25, 2023, *Li JL, Song QR, BNU2023ZJJ01*, (BNU, 2 duplicates); Jinbianxi, 2021, *Zhang C zhang670*, (BNU, 7 duplicates) • Tianmenshan, May 2, 2013, *Zhang DG 130502028* (JIU33520) • Hunan, Exact location unknown, March 18, 1955, *Xia JL 20*, (PE01995438).

##### Discussion.

Major centers of *Cardamine* diversity estimated by the number of taxa appear to be in the European Mediterranean and the Caucasus; Eastern Asia and the Himalayas; and North and Central America (Marhold et al. 2018). Utilizing data from this study, we selected common *Cardamine* in Hunan and Central China, such as *Cardaminelyrata*, *Cardamineanhuiensis*, *Cardamineengleriana*, *Cardaminefragariifolia*, *Cardaminecircaeoides*, *Cardaminescutata*, *Cardaminefallax*, *Cardamineocculta* and determined the phylogenetic position of *C.zhangjiajieensis* within the genus. Phylogenetic analysis shows that *C.zhangjiajieensis* falls in a clade sister to *C.scutata*. From the latter, *C.zhangjiajieensis* is readily distinguished by lateral leaflets 0–2 pairs (vs. 1–4), the terminal leaflet of the cauline leaf 4–7 cm × 3.5–6.5 cm (vs. 2–5 × 1.5–4 cm), petals 4.5–6.5 mm × 2.5–3 mm (vs. 2.5–4.5 × 1.5–2.5 mm), sepals 2.8–3.2 mm × 1.4–1.9 mm (vs. 1.5–2.5 × 0.9–1.4 mm), vegetative propagated by adventitious buds in fruit stage.

As a widespread species in East Asia, the speciation of *C.scutata* has been elucidated. Allotetraploid *C.scutata* originated by hybridization between two diploid species, *C.parviflora* and *C.amara* (Mandáková et al. 2019). Based on chloroplast genome data, our study showed that *C.parviflora*, *C.scutata*, *C.zhangjiajieensis*, *C.amariformis* formed a highly supported subclade, *C.parviflora* located at the base of clade, which supported the results of previous studies. Our analysis also suggested that *C.scutata* (2n = 4x = 32) may be involved in the speciation of *C.zhangjiajieensis* (2n ≈ 6x) as hybrid parent according to the ploidy level.

Another notable character of *C.zhangjiajieensis* is vegetative reproduction by adventitious buds in fruit stage, which is also found in *C.hupingshanensis*, an endemic species to Hupingshan Mountains (Long 2004). From the latter, *C.zhangjiajieensis* is readily distinguished by glabrous (vs. hirsute), terminal leaflet of the cauline leaf oblong, not petiole auriculate-amplexicaul at base (vs. reniform or orbicular, petiole auriculate-amplexicaul at base), seed apically narrow wing (vs. wingless), as shown in Table 4. *C.hupingshanensis* has 2n = 24. In natural conditions, the reproduction of *C.hupingshanensis* relies on adventitious roots growing on each stem node after lodging, indicating that the ploidy level of *C.hupingshanensis* is 2n = 3x. To further clarify speciation and evolution of karyotype within *Cardamine*, studies with comprehensive sampling and seeds germination of *C.zhangjiajieensis* are needed.

**Table 4.** Comparison of characters of *C.zhangjiajieensis*, *C.circaeoides* and *C.scutata*.

	*C.zhangjiajieensis*	*C.circaeoides*	*C.scutata*	*C.hupingshanensis*
Life cycle	Perennial	perennial	annual or biennial	perennial
Trichome	Glabrous	pilose	glabrous or pilose	hirsuta
Plant height	15–40 cm	9–36 cm	(5–)15–50(–70) cm	30–100 cm
Base of petiole	Simple	auriculate	simple	auriculate
No. of lateral leaflet or lobes of the basal leaf	(0–)1–2(–3)	0(–2)	1–4	0(rare 1)
Length of terminal leaflet of the basal leaf	3–5 cm	2–4 cm	1.5–2.5 cm	4–13 cm
Terminal leaflet of the basal leaf	Orbicular	cordate or oval	reniform or cordate	reniform or orbicular
Base of the basal leaf	Truncate	cordate	cordate	cordate, petiole auriculate-amplexicaul at base
Length of terminal leaflet of the cauline leaf	4.5–7 cm	1–3.5 cm	2–5 cm	4–13 cm
Terminal leaflet of the cauline leaf	orbicular to oblong	cordate, ovate to ovate-lanceolate	oblong-oval	reniform or orbicular
Base of the cauline leaf	Ovate	cuneate	ovate	cordate, petiole auriculate-amplexicaul at base
Margin of cauline leaf	undulating sinuses	crenate or subentire	apically sinuses 3–5	crenate
Sepals (mm)	2.8–3.2 × 1.4–1.9	2–3.5 × 0.8–1.5	1.5–2.5 × 0.9–1.4	5–6 × 3–4
Petals (mm)	4.5–6.5 × 2.5–3	5–7 × 2–2.5	2.5–4.5 × 1.5–2.5	8–10 × 7–9
Stamens (mm)	1.8–3	2.5–5	2–3.5	3–6
Ovules of ovary	18–40	20–42	20–40	Not seen
Seed (mm)	1.4–1.6 × 0.9–1.1	0.8–1.1 × 0.6–0.9	0.9–1.2 × 0.6–0.9	1.2–1.8 × 0.9–1.3
Wing of seed	apically narrow wing	wingless	with wing	wingless
(DNA) ploidy level	2n ≈ 6x	2n = 2x = 16	2n = 4x = 32	2n = 3x = 24

**Table 5.** Taxa in phylogenetic analyses.

GenBank accession numbers	Specimens	Species name
NC026446		*Cardamineresedifolia* L.
MT136871		*Cardaminequinquefolia* (M.Bieb.) Schmalh.
MK637691		*Cardaminepentaphyllos* (L.) Crantz
NC036964		*Cardamineparviflora* L.
NC036963		*Cardamineoligosperma* Nutt.
MZ043777		*Cardamineocculta* With.
MF405340		*Cardaminemacrophylla* Willd.
MZ846206		*Cardaminelyrata* Bunge
MK637684		*Cardaminekitaibelii* Bech.
NC026445		*Cardamineimpatiens* L.
ON322745		*Cardaminehupingshanensis* K.M.Liu
MK637681		*Cardaminehirsuta* L.
MN651504		*Cardamineheptaphylla* (Vill.) O.E.Schulz
MK637680		*Cardamineglanduligera* O.Schwarz
MZ043778		*Cardaminefallax* L.
NC049605		*Cardamineenneaphyllos* (L.) Crantz
OL634846		*Cardaminecircaeoides* Hook.f. & Thomson
NC049603		*Cardaminebulbifera* (L.) Crantz
MN651509		*Cardaminebipinnata* (C.A.Mey.) O.E.Schulz
MZ043776		*Cardamineamariformis* Maxim.
NC036962		*Cardamineamara* L.
NC060863		*Cardamineabchasica* Govaerts
KJ136821		*Cardamineimpatiens* L.
NC069649		*Rorippasylvestris* (L.) Besser
NC065833		*Rorippaindica* (L.) Hiern
PP114745	*ZJJ02*, Li JL, Zhang C	*Cardaminezhangjiajieensis*
PP114744	*Msc02*, Li JL, Zhang C	*Cardaminescutata* Thunb.
PP719683	*Zhang549*, Zhang C	*Cardamineanhuiensis* D.C.Zhang & C.Z.Shao
PP719686	*Zhang664*, Zhang C	*Cardaminehygrophila* T.Y.Cheo & R.C.Fang
PP719685	*EM105*, Li JL, Zhang C	*Cardaminefragariifolia* O.E.Schulz
PP719684	*Msc05*, Li JL, Zhang C	*Cardamineengleriana* O.E.Schulz

## Supplementary Material

XML Treatment for Cardamine zhangjiajieensis

## ﻿Additional information

### Author ORCIDs

Jia-Lu Li https://orcid.org/0009-0002-8147-5157

Cheng Zhang https://orcid.org/0000-0003-1020-0850

Yi He https://orcid.org/0000-0002-6925-7299

### Data availability

All of the data that support the findings of this study are available in the main text.
